# Synergistic effect of Er, Cr: YSGG laser and nano-hydroxyapatite on the remineralization of initial enamel lesions: a microhardness, surface roughness and SEM analysis

**DOI:** 10.1186/s12903-026-08597-9

**Published:** 2026-05-26

**Authors:** Ecehan Kaplan, Sergen Özdemir, Ayşe Dündar, Çağatay Barutçugil, Edip Bayram

**Affiliations:** 1 Department of Restorative Dentistry, Faculty of Dentistry, Antalya Bilim University, Antalya, 07040 Türkiye; 2 Private Practice, Antalya, Türkiye; 3 Department of Restorative Dentistry, Faculty of Dentistry, DDS, Akdeniz University, Antalya, Türkiye; 4 Department of Chemistry, Institute of Natural and Applied Sciences, Akdeniz University, Antalya, Türkiye Turkey

**Keywords:** Enamel, Er, Cr:YSGG laser, Microhardness, Nano-hydroxyapatite, Remineralization, Surface roughness

## Abstract

**Background:**

Initial enamel lesions represent a reversible stage of dental caries, yet conventional remineralization methods often face challenges in acid resistance. This study aimed to evaluate the synergistic effect of Er, Cr: YSGG laser and nano-hydroxyapatite (nano-HA) on the remineralization of initial enamel lesions and their sustainability against subsequent acid challenges.

**Methods:**

One hundred bovine incisors were used to create artificial white spot lesions. Specimens were randomly assigned to five groups (*n* = 20): G1 (CPP-ACP + F), G2 (Nano-HA), G3 (Er, Cr: YSGG laser), G4 (Laser + Nano-HA), and G5 (Nano-HA + Laser). After treatment, specimens underwent a 10-day pH-cycling protocol (6 h demineralization/18 h artificial saliva). Surface microhardness (Vickers), surface roughness (Ra), and surface morphology (SEM) were evaluated at three stages: post-demineralization, post-remineralization, and post-pH-cycling. Data were analyzed using Two-way and Repeated Measures ANOVA with Tukey HSD post-hoc tests (*p* < 0.05).

**Results:**

Laser-assisted groups (G3, G4, and G5) exhibited significantly higher microhardness values compared to G1 and G2 across all stages (*p* < 0.05). Following pH-cycling, G4 (Laser + Nano-HA) showed significantly higher hardness preservation compared to G5 (Nano-HA + Laser) (*p* < 0.05). While laser application significantly increased surface roughness in all measurement stages (*p* < 0.05), the application of nano-HA in G4 and G5 partially masked these irregularities. SEM analysis confirmed that G4 provided a more compact and homogeneous mineral layer, with fewer longitudinal cracks after acid challenge compared to other groups.

**Conclusions:**

The combination of Er, Cr: YSGG laser and nano-HA, particularly when the laser is applied first, significantly enhances the remineralization efficacy and acid resistance of enamel. This synergistic approach offers a promising clinical alternative for the long-term management of initial caries lesions.

**Supplementary Information:**

The online version contains supplementary material available at 10.1186/s12903-026-08597-9.

## Background

Enamel demineralization is a condition characterized by the dissolution of mineral content within the dental hard tissues, frequently manifesting as initial caries lesions, also known as white spot lesions [1]. These lesions lead to not only aesthetic concerns but also functional impairments [2]. Nevertheless, since these lesions exhibit reversible characteristics during their early stages, remineralization strategies have emerged as a significant therapeutic option in clinical practice [3].

The most common strategy for remineralization involves fluoride applications. Fluoride enhances the acid resistance of the enamel surface by promoting the formation of fluorapatite [4]. In addition to fluoride, alternative remineralizing agents, either fluoride-free or combined with fluoride, are also utilized. Among these materials, casein phosphopeptide-amorphous calcium phosphate (CPP-ACP) stabilizes calcium and phosphate ions, facilitating their transport to the demineralized enamel surface [5]. Furthermore, the concomitant application of CPP-ACP with fluoride may exhibit a synergistic effect, enhancing the integration of fluoride into the crystal lattice [6].

Another prevalent approach is the use of nano-hydroxyapatite (nano-HA) due to its bioactive and biomimetic properties. Owing to its crystalline structure similar to natural enamel and its nano-scale particle size, nano-HA possesses a high affinity for demineralized areas. Its mechanism primarily occurs through two pathways: first, the nano-particles physically fill the microporosities on the enamel surface, creating a sealing effect and increasing surface microhardness [7]. Second, it releases calcium and phosphate ions in acidic environments, increasing the local ion concentration and promoting crystal growth (remineralization) [8]. Studies have demonstrated that nano-HA provides more effective results in compensating for mineral loss and reducing surface roughness in initial enamel lesions compared to conventional methods [8, 9].

Laser technologies are also being investigated for the management of early enamel lesions due to their photothermal interaction potential. Specifically, the Er, Cr: YSGG laser (2780 nm) is highly absorbed by the water and hydroxyapatite crystals within the enamel structure. This interaction rapidly increases the temperature on the enamel surface, leading to a reduction in the carbonate content of hydroxyapatite crystals and an increase in crystal size. This transforms the enamel into a ‘recrystallized’ structure that is more resistant to acid dissolution [10]. Furthermore, it is suggested that the sub-ablative micromorphological changes induced by laser application may provide an ideal mechanical interlocking area for subsequently applied remineralizing agents, thereby enhancing their penetration and efficacy [11].

In recent years, in vitro studies exploring the potential synergistic effects of combining laser applications with agents such as CPP-ACP or nano-HA have reported that the roughened surface prepared by the laser increases the retention of nano-particles [12]. However, findings in the literature remain inconsistent due to methodological differences such as laser parameters, application sequences, and material types; thus, more controlled in vitro studies are required [13].

The aim of this study was to compare the remineralization efficacy of CPP-ACP with fluoride (MI Paste Plus), nano-HA, and Er, Cr: YSGG laser applications, both individually and in combination, on artificially demineralized enamel surfaces. In this context, the null hypotheses of the study were defined as follows:

### H0₁

There is no statistically significant difference in the remineralization efficacy among the different treatment protocols.

### H0₂

There is no statistically significant difference among the different treatment protocols in their capacity to maintain remineralization gains following an acid challenge.

### H0₃

There is no statistically significant difference between Laser→nano-HA and nano-HA→Laser applications in terms of remineralization efficacy and the sustainability of this effect.

## Methods

### Preparation of specimens

Based on the G*Power analysis (alpha = 0.05, power = 0.80), a total of 100 bovine mandibular incisors were used, with 20 specimens allocated to each group [14]. After removing residual tissues, teeth that were caries-free and devoid of crown defects were stored in a 0.5% chloramine-T solution.

**Fig. 1:**
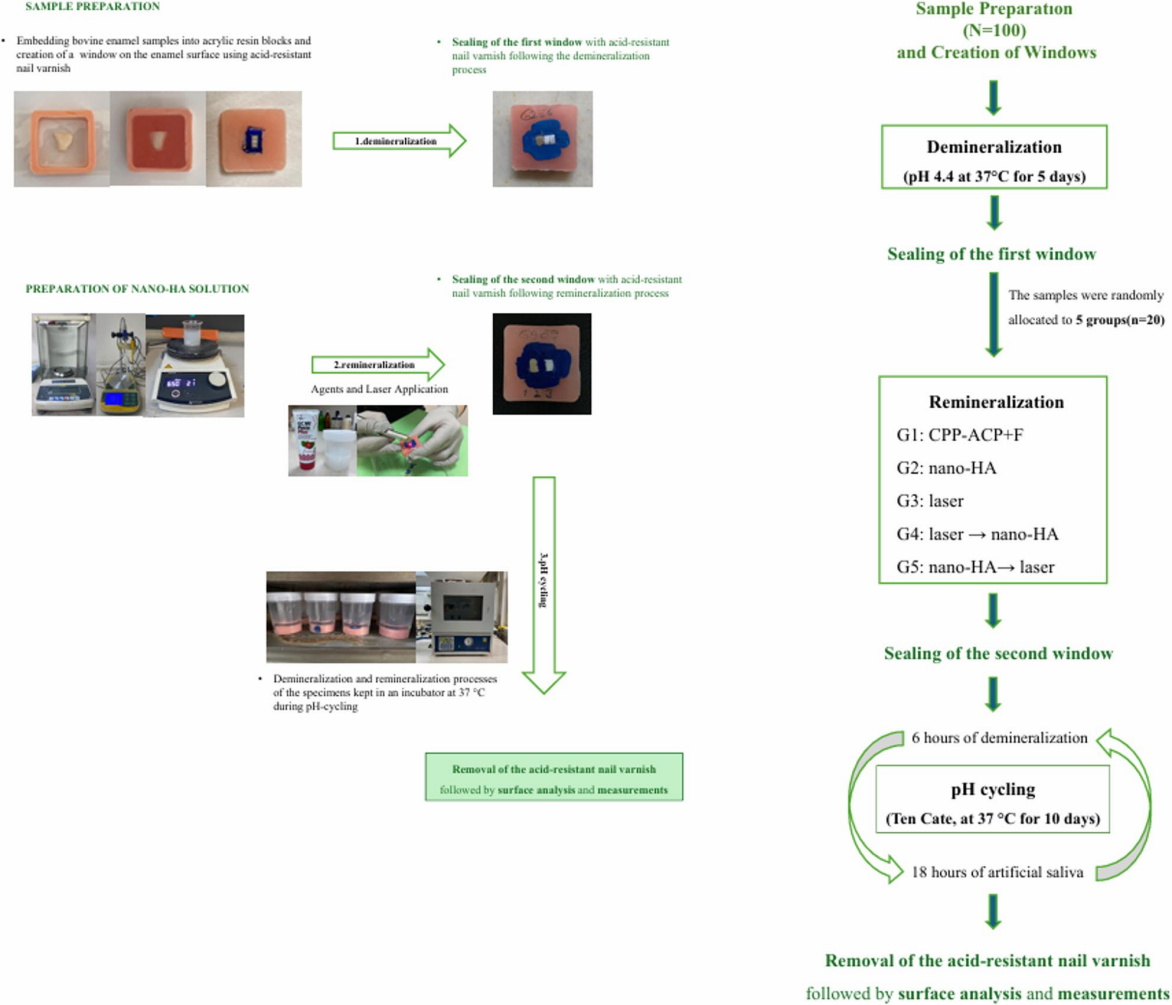
Flowchart of the experimental study workflow. The diagram illustrates the sequential stages of the study, including specimen preparation, group allocation, remineralization protocols, and the pH-cycling process

The crowns were separated from the cemento-enamel junction using a precision cutting device (Isomet Low-speed, Buehler, Illinois, USA) under water cooling. The crowns were then embedded in autopolymerizing acrylic resin, leaving the vestibular surfaces exposed (Fig. 1). To obtain a flat surface while preserving the enamel tissue, the specimens were polished with 800-, 1200-, and 2400-grit silicon carbide abrasive papers (Metkon, Bursa, Turkey). An acid-resistant nail varnish was applied to the enamel surface to define a window of approximately 6 × 2 mm, which was further divided into three equal areas using a pencil (Fig. 1).

### Preparation of Nano-HA

Nano-hydroxyapatite was synthesized using the sol-gel technique [15]. Solution A was prepared by adding 8.8 g of calcium phosphate monobasic (CaH₄(PO₄)₂) into 3.17 mL of distilled water and stirring until homogeneous. For Solution B, the pH value was adjusted to 10 by slowly adding ammonia into phosphoric acid. Then, 25 mL of Solution B was added dropwise to Solution A, and the mixture was stirred at high speed for 30 min. Subsequently, 10 g of calcium hydroxide was added to the mixture, which was then agitated in an ultrasonic vibrator for 1 h. The resulting slurry was aged at room temperature for 24 h and then dried at 65 °C for another 24 h. The dried gel was washed with distilled water and filtered to remove ammonium phosphate. Following this, the sludge-like mixture was subjected to a sintering process by gradually heating from 200 °C to 800 °C. Finally, the sintered HA sample was pulverized into a fine powder.

The chemical reactions for the synthesized nano-HA are as follows:

$${\mathrm H}_3{\mathrm{PO}}_4+3{\mathrm{NH}}_4\mathrm{OH}\rightarrow{\left({\mathrm{NH}}_4\right)}_3{\mathrm{PO}}_4+3{\mathrm H}_2\mathrm O$$

$${{\mathrm{CaH}}_4{\left({\mathrm{PO}}_4\right)}_2\left(\mathrm s\right)}\rightarrow\mathrm{Ca}{\left({\mathrm H}_2{\mathrm{PO}}_4\right)}_2\left(\mathrm s\right)$$

$$3\mathrm{Ca}{\left({\mathrm H}_2{\mathrm{PO}}_4\right)}_2+2{\left({\mathrm{NH}}_4\right)}_3{\mathrm{PO}}_4\rightarrow{\mathrm{Ca}}_3{\left({\mathrm{PO}}_4\right)}_2+6{\mathrm{NH}}_4{\mathrm H}_2{\mathrm{PO}}_4$$

$$3{\mathrm{Ca}}_2{\left({\mathrm{PO}}_4\right)}_2+\mathrm{Ca}{\left(\mathrm{OH}\right)}_2\rightarrow{\mathrm{Ca}}_{10}{\left({\mathrm{PO}}_4\right)}_6{\left(\mathrm{OH}\right)}_2$$

**Fig. 2:**
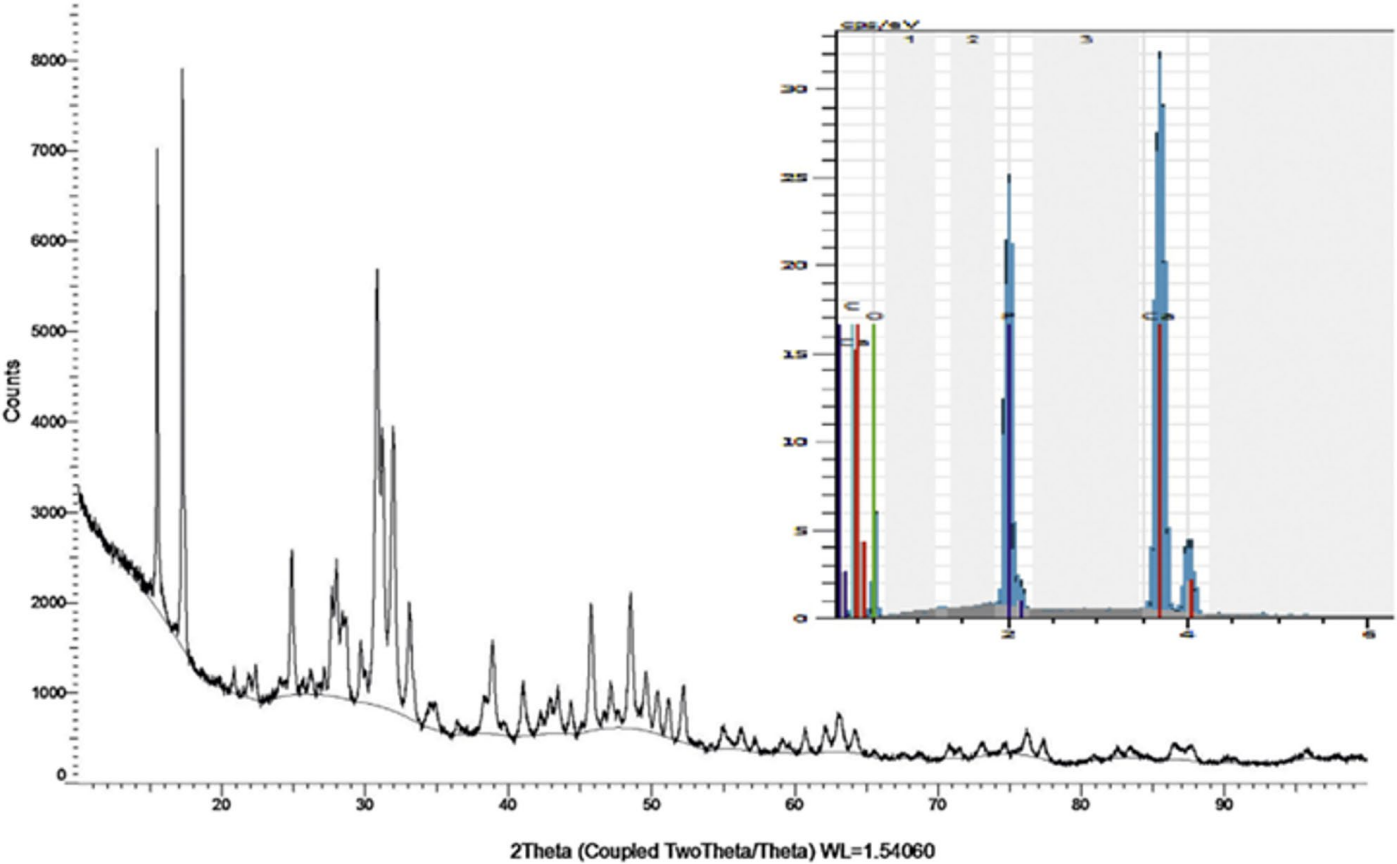
X-Ray diffraction pattern (left) and energy-dispersive X-ray spectroscopy (EDS) spectrum (right) of synthesized nano-hydroxyapatite powder

The elemental analysis of the HA powders was performed using Energy Dispersive X-ray Spectroscopy (EDS), and the crystalline structure was examined via X-ray Diffraction (XRD) (Fig. 2). The average crystallite size (D) was calculated as 30 nm according to the Scherrer equation (Eq. 1): D = Kλ/ βcosθ where: K is the dimensionless shape factor (commonly 0.9), λ is the X-ray wavelength (e.g., 1.5406 Å for CuKα), β is the line broadening at full width at half maximum (FWHM) in radians, θ is the Bragg angle.

### Preparation of Nano-HA Solution

The working solution of the synthesized nano-HA powder was prepared according to the method described by Besinis et al. [16]. For this purpose, nano-HA powder at a concentration of 0.3% (w/v) was added to distilled-deionized water. To minimize agglomeration, 0.05% sodium hexametaphosphate (SHMP) was added to the mixture, and the pH was maintained within the range of 6.8–7.4. The mixture was homogenized in an ultrasonic vibrator for 20–30 min. Finally, 99% pure glycerol was added to achieve a cream-like consistency, preparing the mixture for application [16] (Fig. 1).

### Demineralization protocol

Artificial caries-like lesions were created following the protocol proposed by Pulido et al. [17]. The specimens were individually immersed in a demineralization solution (30 mL/specimen at 37 °C), which was refreshed daily. The composition of the demineralization solution was as follows: 2.2 mM CaCl_2_·2H_2_O, 2.2 mM KH_2_PO_4_, and 0.05 M acetic acid, with the pH adjusted to 4.4 using 10 M KOH. The tooth specimens were kept in this solution for 5 days to ensure the formation of homogeneous white spot lesions on the enamel surface [18]. Subsequently, the enamel specimens were carefully rinsed, and the first window was sealed with acid-resistant nail varnish to serve as a baseline for comparison with the demineralized area during the remineralization phase. The remaining two windows were subjected to the remineralization protocols described below.

### Group allocation and remineralization protocol

The specimens were randomly assigned to five experimental groups (*n* = 20 per group, total *n* = 100):

#### G1 (CPP-ACP + F)

0.05 g of CPP-ACP + F (MI Paste Plus, GC Corp., Tokyo, Japan) was applied to the enamel surface using a microbrush until completely covered. The paste was left for 5 min, and the excess was removed with a cotton pellet.

#### G2 (nano-HA)

0.05 g of the synthesized Nano-HA was applied with a microbrush, left for 5 min, and the excess was removed with a cotton pellet.

**G3 (Er**,** Cr: YSGG laser)**: Er, Cr: YSGG laser (Waterlase, Biolase, California, USA) was applied with the following parameters: 0.5 W power, 20 Hz frequency, 2780 nm wavelength, 25 mJ pulse energy, 60 µs pulse duration, and an energy density of 8.84 J/cm^2^. Cooling was provided by a 60% water–40% air mixture. An MZ6 tip with a 600 μm diameter was held perpendicular to the enamel surface at a distance of approximately 1–1.5 mm, and the laser was applied for a total of 15 s.

#### G4 (Laser + nano-HA)

Following the laser application as described above, 0.05 g of nano-HA was applied with a microbrush, left for 5 min, and the excess was removed with a cotton pellet.

#### G5 (nano-HA + laser)

0.05 g of nano-HA was applied with a microbrush and left for 5 min. After removing the excess with a cotton pellet, laser irradiation was performed using the parameters mentioned above.

Following the remineralization protocols, the second enamel window was sealed with acid-resistant nail varnish.

### pH-Cycling

To realistically simulate the clinical demineralization–remineralization processes, the Ten Cate pH-cycling model was applied to the third enamel window. In this model, a demineralization solution (pH 4.5) containing 2.2 mM Ca, 2.2 mM phosphate, and 50 mM acetic acid/potassium acetate was utilized [3].

The specimens were subjected to pH-cycling for 10 days in an incubator at a constant temperature of 37 °C. The cycle consisted of 6 h of immersion in the demineralization solution followed by 18 h in an artificial saliva solution (formulated by Torres et al.) [19]. All solutions were refreshed every two days (Fig. 1).

Three measurement windows were established to allow for comparisons: the first surface (post-demineralization), the second surface (post-remineralization), and the third surface (post-pH-cycling).

### Surface microhardness measurement

Following demineralization, remineralization, and pH-cycling, the nail varnish on the enamel windows was gently removed using cotton pellets soaked in acetone. The brief application of acetone was performed solely to dissolve the organic nail varnish, which does not interfere with the inorganic mineral structure of the underlying enamel. The surface microhardness (SMH) of each specimen was measured using a microhardness testing machine (HMV-2, Shimadzu, Tokyo, Japan) equipped with a Vickers diamond indenter (100 g load, 5 s dwell time). Three indentations were made at different points within each designated measurement window, and the mean value was used for statistical analysis.

### Surface roughness measurement

A mechanical profilometer (Surftest SJ-201k, Mitutoyo, Kawasaki, Japan) was used to assess the surface roughness of the specimens. The device was calibrated before the start of the study and prior to measuring each group (λc: 0.8; λs: 2.5). Three measurements were taken from different locations on each specimen, and the average surface roughness value (Ra, µm) was calculated by averaging these data points.

### Surface morphology analysis

**Fig. 3:**
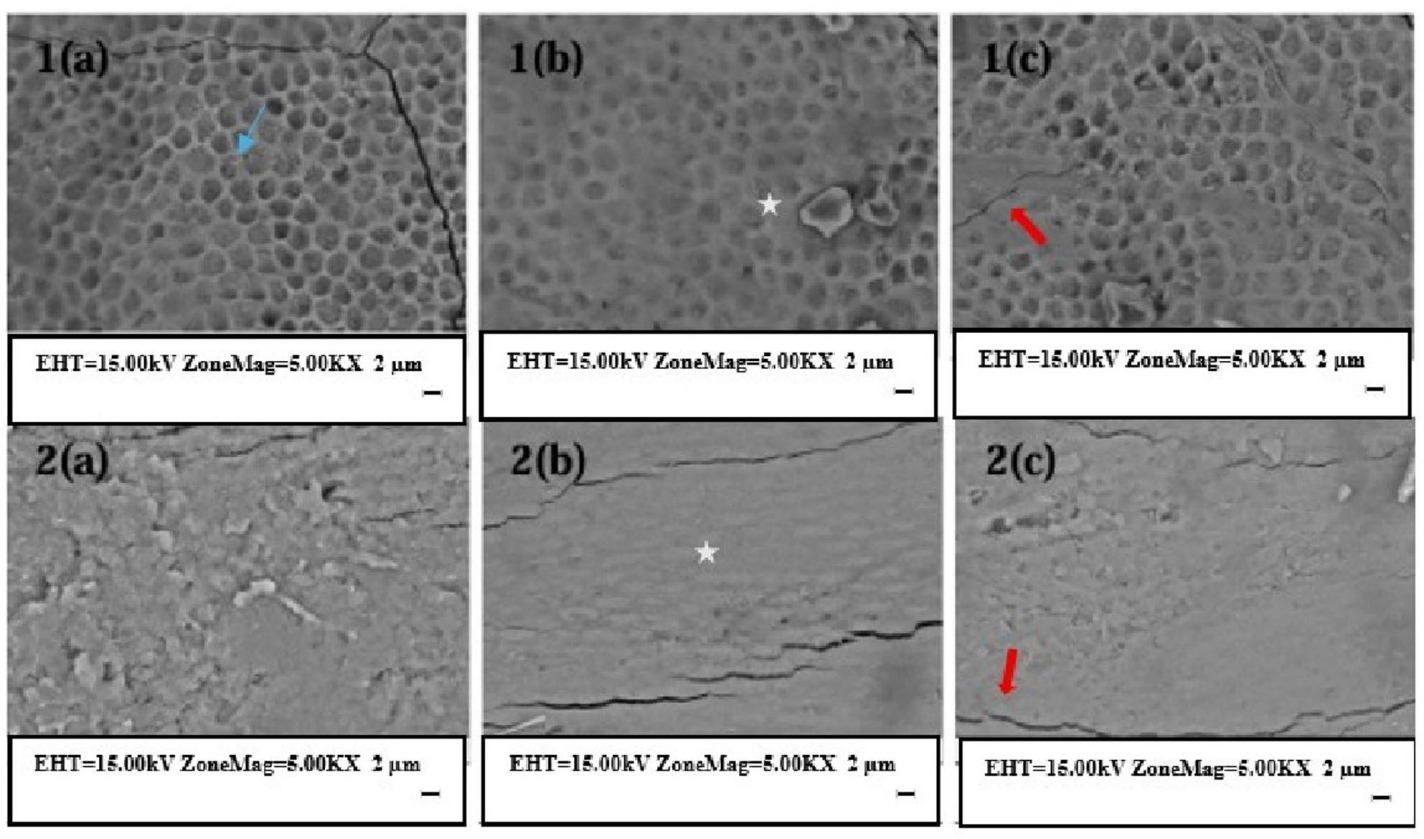
SEM micrographs of non-laser groups (G1: CPP-ACP + F and G2: Nano-HA) at 5,000x magnification. (**a**) After demineralization: Surface morphology displaying the characteristic honeycomb pattern and increased interprismatic spaces (blue arrows). (**b**) After remineralization: Surface showing the deposition of mineral precipitates within the demineralized areas, resulting in a more regular topography (asterisks) (**c**) After pH-cycling: Re-emergence of surface irregularities and appearance of longitudinal cracks (red arrows) resulting from the repeated acid challenge. Scale bars represent 2 μm

The surface morphology of selected specimens was examined under a scanning electron microscope (SEM; JEOL JSM-5600LV, Tokyo, Japan) at 5,000× magnification.(Figure 3) Before SEM analysis, the specimen surfaces were rendered conductive by sputter-coating with 99.9% pure gold. Comparative images were obtained for the surfaces post-demineralization, post-remineralization, and post-pH-cycling.

### Statistical analysis

Data were expressed as mean ± standard deviation. The normality of the data distribution was assessed using the Shapiro-Wilk test. Two-way Analysis of Variance (Two-way ANOVA) or Repeated Measures ANOVA was utilized to analyze the differences between groups (inter-group) and between different surfaces within the same group (intra-group). Multiple comparisons were performed using the Tukey HSD post-hoc test. The level of statistical significance was set at *p* < 0.05 (SPSS v16.0, SPSS Inc., Chicago, IL, USA).

## Results

### Surface microhardness

When the microhardness values were examined during the demineralization phase, no statistically significant differences were observed among the G1, G2, and G3 groups (*p* > 0.05). However, the combination groups of laser and nano-HA (G4 and G5) exhibited significantly higher baseline hardness compared to the G1 group (*p* < 0.05) (Table 1).

**Table 1:** Mean surface microhardness (Vickers) values and standard deviations of the experimental groups

Groups	Surface 1 (Post-demineralization)	Surface 2 (Post-remineralization)	Surface 3 (Post-pH-cycling)
**G1**	101.95 ± 23.4 ^a, A^	125.94 ± 40.39 ^a, B^	116.5 ± 11.6 ^a, B^
**G2**	110.22 ± 84.76 ^ab, A^	147.19 ± 54.98 ^a, A^	124.33 ± 49.56 ^a, A^
**G3**	151.31 ± 66.45 ^abc, A^	223.16 ± 78.15 ^b, B^	178.66 ± 61.3 ^b, B^
**G4**	153.98 ± 29.33 ^bc, A^	245.81 ± 85.48 ^b, B^	201.61 ± 49.12 ^b, B^
**G5**	161.77 ± 63.98 ^c, A^	240.15 ± 77.66 ^b, B^	183.31 ± 67.54 ^b, A^

According to the results of One-way analysis of variance and Tukey HSD multiple comparison test, lowercase letters in the columns and uppercase letters in the rows indicate statistical differences between groups(*p* < 0.05)

Following the application of different treatment protocols, an increase in enamel microhardness was observed across all groups. Statistical analysis revealed that the groups involving laser application (G3, G4, and G5) exhibited significantly higher microhardness values compared to the non-laser groups (G1 and G2) (*p* < 0.05). No statistically significant difference was found between the CPP-ACP + F and nano-hydroxyapatite groups in terms of microhardness values (*p* > 0.05). Furthermore, the sequence of laser and nano-HA application (Laser + HA vs. HA+Laser) did not demonstrate a statistically significant superiority regarding early-stage hardness gain (*p* > 0.05).

At the end of the pH-cycling phase, groups G3 and G4 (laser-involved protocols) showed significantly higher microhardness values compared to the non-laser methods (G1 and G2) (*p* < 0.05). At this stage, a statistically significant difference was identified between the combined laser and nano-hydroxyapatite groups; specifically, group G4 (laser application followed by nano-HA) exhibited higher microhardness values than group G5 (nano-HA application followed by laser) (*p* < 0.05) (Table 1).

### Surface roughness

While initial demineralization and pH-cycling increased Ra values in all groups, the laser-involved groups (G3, G4, and G5) demonstrated significantly higher surface roughness values compared to the CPP-ACP + F and nano-hydroxyapatite groups across all measurement stages (*p* < 0.05) (Table 2).

**Table 2:** Mean surface roughness (Ra, µm) values and standard deviations of the experimental groups

Groups	Surface 1 (Post-demineralization)	Surface 2 (Post-remineralization)	Surface 3 (Post-pH-cycling)
**G1**	2.65 ± 1.12 ^a, A^	1.3 ± 0.75 ^a, B^	2.3 ± 1.1 ^a, A^
**G2**	2.05 ± 1.05 ^a, A^	1.64 ± 0.74 ^ab, A^	2.12 ± 0.69 ^a, A^
**G3**	4.37 ± 0.9 ^b, A^	2.15 ± 0.86 ^bc, B^	4.68 ± 1.52 ^c, A^
**G4**	3.96 ± 1.53 ^b, A^	2.48 ± 0.68 ^c, B^	3.47 ± 1.37 ^b, A^
**G5**	4.09 ± 0.83 ^b, A^	2.3 ± 0.68 ^c, B^	3.68 ± 0.97 ^b, A^

According to the results of one-way analysis of variance and Tukey HSD multiple comparison test, lowercase letters in the columns and uppercase letters in the rows indicate statistical differences between groups(*p* < 0.05)

The application of remineralizing materials significantly reduced surface roughness in all groups (*p* < 0.05). Group G1 showed the statistically lowest Ra values compared to groups G3, G4, and G5. The combination groups, G4 and G5, exhibited similar results in terms of roughness values, with no statistically significant difference between them (*p* > 0.05) (Table 2).

### Surface morphology findings (SEM Analysis)

In the SEM images corresponding to the initial demineralization stage, all groups exhibited morphological alterations associated with mineral loss. However, due to the inherent anisotropic structure of bovine enamel and the varying orientation of enamel prisms relative to the surface, the demineralization patterns showed visual variations across specimens. While some areas displayed a distinct honeycomb pattern reflecting mineral loss in the central regions of the enamel prisms **(**Figs. 1a and 3, blue arrow), other areas exhibited more superficial roughness and morphological irregularities with widespread microporosities. Despite these visual variations, extensive demineralization was confirmed across all specimens.

**Fig. 4:**
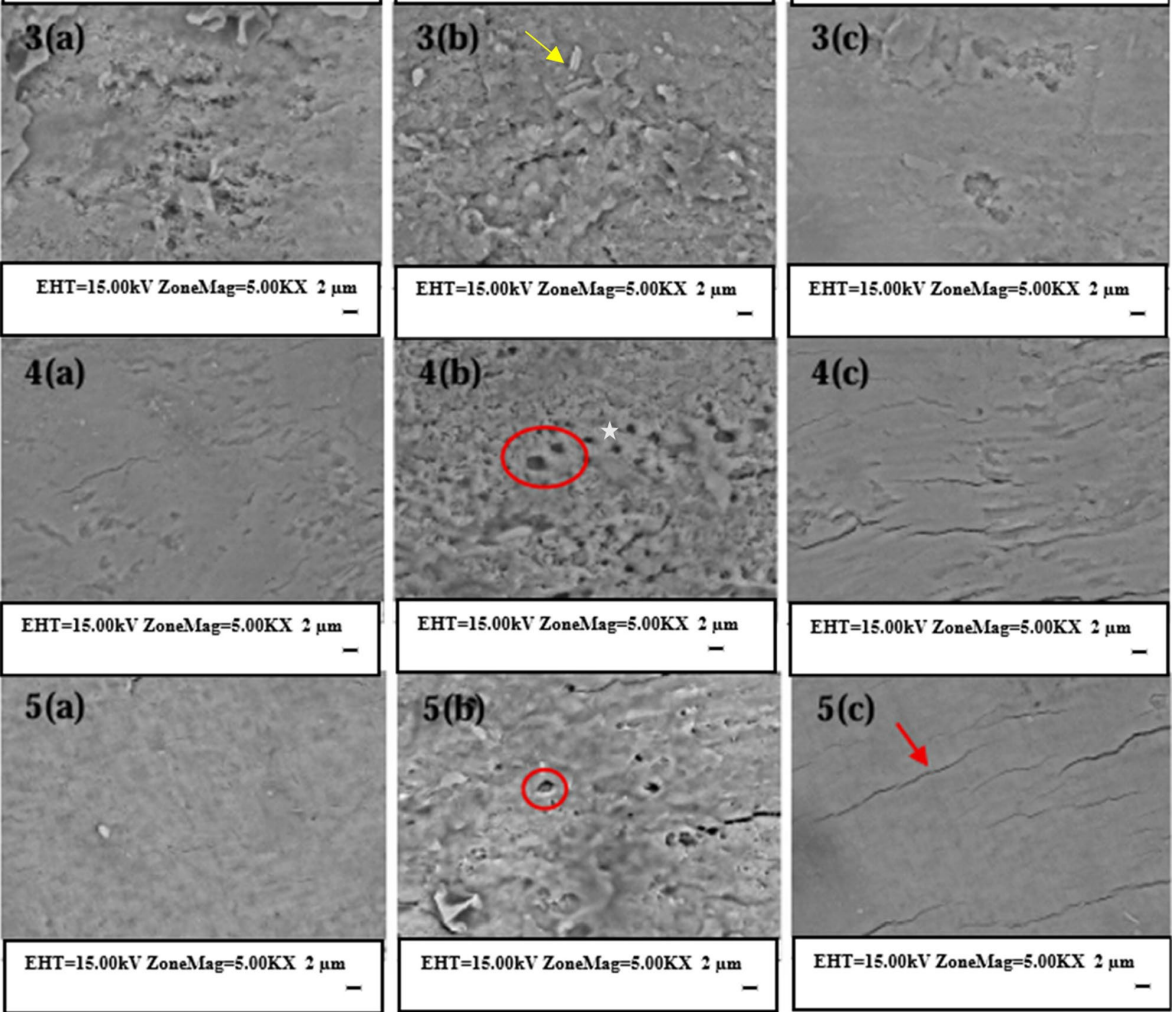
SEM micrographs of laser-assisted groups (G3: Laser only, G4: Laser + Nano-HA, and G5: Nano-HA + Laser) at 5,000x magnification. (**a**) After demineralization: Baseline demineralized enamel morphology. (**b**) After remineralization: Structural modifications including localized melting points (yellow arrow in 3b) and micro-crater formations (red circles in 4b and 5b). In Group G4, the nano-particles are seen effectively “sealing” the laser-induced micro-porosities (asterisk in 4b). (**c**) After pH-cycling: While surface integrity is largely preserved, the appearance of longitudinal cracks (red arrow in 5c) indicates the impact of repeated acid challenges. Scale bars represent 2 μm

Following the remineralization treatments, surface morphology varied depending on the agent applied. In the non-laser groups (G1: CPP-ACP + F and G2: Nano-HA), mineral precipitates were observed to partially occlude the demineralized pores, resulting in a smoother surface topography **(**Figs. 2b and 3, asterisks). Conversely, the groups treated with the Er, Cr: YSGG laser (G3, G4, and G5) displayed distinct structural modifications resulting from the photothermal effect. These included localized melting points **(**Figs. 3b and 4, yellow arrow) and micro-crater formations **(**Figs. 4 and b and 5b, red circles). Notably, in Group G4, the nano-particles effectively integrated into and “sealed” these laser-induced micro-porosities, forming a denser and more compact mineral layer **(**Fig. 4 and b, white asterisk).

Examination after pH-cycling revealed signs of acid-induced wear on the surfaces of all groups. While surface destruction and the re-emergence of the prismatic structure were more widespread in the non-laser groups **(**Figs. 1c, 2c and 3**)**, the laser-assisted protocols were found to preserve surface integrity more effectively. In comparing the application sequences, Group G4 (laser followed by nano-HA) exhibited more homogeneous surface continuity compared to Group G5 (nano-HA followed by laser), where longitudinal cracks resulting from acid erosion were more prominent **(**Figs. 4 and 5c, red arrow).

## Discussion

This in vitro study provided a comprehensive evaluation of the effects of various remineralization protocols on initial enamel lesions through microhardness testing, surface roughness measurement, and SEM analysis. The primary finding of the study is that laser-assisted remineralization applications (G3, G4, and G5) exhibited statistically significantly higher Vickers microhardness values compared to conventional CPP-ACP + F (G1) and nano-HA (G2) groups, both during the early remineralization phase and after the subsequent acid challenge. In light of these data, the null hypotheses H0₁ and H0₂ were rejected, as significant differences were identified among the treatment protocols regarding their remineralization efficacy and their capacity to maintain mineral gains following an acid challenge. The microhardness results of the present study are consistent with the literature reported by Adel et al., which demonstrated that the combination of Er, Cr: YSGG laser and CPP-ACP exhibits a superior synergistic effect compared to the individual use of these methods [20].

Similarly, Patil et al. reported that the use of laser radiation in conjunction with nano-HA significantly enhanced microhardness compared to the use of these agents alone. The high Vickers values obtained in our study confirm that the laser functions as a priming and reinforcing mechanism for remineralizing agents, which aligns with existing literature [21].

The preference for bovine incisors to evaluate enamel lesions in our study is due to the fact that these teeth exhibit a histological structure and chemical composition similar to human enamel. Furthermore, they provide ease of standardization under laboratory conditions thanks to their large and uniform surface areas [17].

The five-day demineralization protocol used to create initial enamel lesions allowed for the acquisition of homogeneous and reproducible ‘white spot lesions.’ However, to more realistically simulate the dynamic demineralization-remineralization processes occurring in the clinical environment, a 10-day pH-cycling period based on the Ten Cate model was implemented following the remineralization phase [3]. This cycle provided a foundation for evaluating the H0₂ hypothesis by testing not only the treatment efficacy but also the sustainability of the remineralized tissue against subsequent acid challenges.

The fundamental effect of the Er, Cr: YSGG laser on enamel tissue is to induce structural modification in enamel crystals through photothermal interaction. The high microhardness values exhibited by the laser-treated groups (G3, G4, and G5) in our study can be explained by the laser energy increasing the size of hydroxyapatite crystals, reducing carbonate content, and transforming the tissue into a “recrystallized” structure that is more resistant to acid dissolution. As stated by Bakhtiary et al., laser radiation improves the enamel crystal structure, stabilizes ion exchange, and creates a scaffold for remineralizing agents [13, 22]. On the other hand, the significantly higher surface roughness observed in the laser groups (G3, G4, and G5) compared to other groups in Table 2 is a natural consequence of the Er, Cr: YSGG laser creating micro-explosions on the enamel surface, thereby roughening the tissue at a microscopic level. As reported by Tang et al., this effect of erbium lasers permanently alters the enamel surface morphology [23–25]. However, our results suggest that these morphological irregularities, rather than being a disadvantage, enhance remineralization success by creating an ideal area for mechanical interlocking and entrapment for nano-HA particles, as emphasized by Alajlan et al. [26].

When examining the non-laser remineralization protocols, namely the CPP-ACP + F (G1) and nano-HA (G2) groups, both materials were found to provide statistically similar levels of microhardness increase in initial enamel lesions (Table 1). The hardness increase in the MI Paste Plus (G1) group can be attributed to casein phosphopeptides stabilizing calcium and phosphate ions to create an ion reservoir on the demineralized enamel surface, synergistically promoting fluorapatite formation with the 900 ppm fluoride in its formulation [27]. Conversely, the remineralization success observed in the nano-HA (G2) group can be linked to the material’s biomimetic similarity to natural enamel tissue and its ability to compensate for mineral loss due to the high surface-to-volume ratio provided by its nano-scale particle size [7]. However, the fact that both groups lagged behind the laser-assisted protocols after remineralization is related to the mechanism of mineral deposition. Huang et al. reported that when nano-HA particles are applied alone, they tend to accumulate mostly in the outermost layer of the enamel, which may limit the diffusion of ions into the deeper layers of the lesion [9]. In our study, the loss of hardness experienced by the G1 and G2 groups following the acid challenge can be explained by the resulting mineral layer not being as sealed or deeply integrated as in the laser groups, thus leaving the acid resistance more limited. This supports the theory proposed by Patil et al. that while nano-HA is effective as a surface filler on its own, it cannot reach maximum stability without the micromorphological infrastructure prepared by the laser [21].

One of the most distinctive findings of our study is the impact of the application sequence of remineralization protocols on enamel surface characteristics. Following pH-cycling, it was determined that Group G4 (laser followed by nano-HA) maintained statistically significantly higher microhardness values compared to Group G5. Conversely, when examining surface roughness values, no statistically significant difference was found between the G4 and G5 groups across all measurement stages (*p* > 0.05). This indicates that while the application sequence plays a critical role in microhardness gain and acid resistance, it does not induce a similar change in the overall roughness character of the surface. Due to this complex dataset, the H0₃ hypothesis, which posits that the application sequence makes no difference, was rejected regarding the microhardness parameter but accepted in terms of surface roughness.

The superiority of the G4 group in microhardness can be explained by the theory proposed by Adel et al., which suggests the sealing of laser-induced micro-voids by remineralizing agents [20]. The micro-craters created by the Er, Cr: YSGG laser provided an ideal attachment area for the subsequently applied nano-HA particles; as noted by Alajlan et al., the entrapment of these particles within porosities stabilized the mineral precipitate [26]. The finding that roughness values were similar in both groups may be attributed to the applied nano-HA solution masking the laser-induced macro-irregularities in a similar fashion. However, in Group G5, applying nano-HA before the laser may have caused the mineral particles to be ablated by the laser’s thermal effect or hindered their sufficient integration into micro-cracks, thereby weakening acid resistance. The more homogeneous surface continuity exhibited by the G4 group in SEM images morphologically proves that the mineral layer in G4 was more effectively integrated into the tissue, even though the roughness values were numerically similar.

In the present study, all groups treated with the Er, Cr: YSGG laser (G3, G4, and G5) demonstrated statistically significantly higher roughness values compared to the non-laser groups (G1 and G2) (*p* < 0.05). This finding is in complete agreement with the data from Tang et al., who reported that erbium lasers increase surface roughness as an expected outcome on the enamel surface [25]. Although an increase in Ra values on the enamel surface is clinically considered a factor that may increase the risk of bacterial adhesion and plaque accumulation, the increase in microhardness and acid resistance obtained in our study is of a nature that can offset this disadvantage. As emphasized by Patil et al., the fact that nano-HA reconstructs the surface by repairing the laser-induced rough areas like a filler proves that morphological irregularities can be utilized in favor of remineralization [21]. Furthermore, it is thought that a precision polishing step following laser-assisted remineralization in clinical applications could both preserve the gained hardness and minimize surface roughness.

The limitations of our study involve the inability to fully simulate dynamic factors such as salivary flow rate, microbial flora variations, masticatory forces, and thermal changes within the in vitro design. Furthermore, the depth of the demineralized lesions and the mineral density distribution were not quantified using gold-standard methods such as transverse microradiography (TMR) or micro-computed tomography (micro-CT) [28, 29]. Instead, the formation of white spot lesions was confirmed through established protocols, visual inspection, and microhardness reduction [30]. Future studies should investigate the long-term clinical follow-up of the laser and nano-HA combination, as well as the effects of different laser parameters (power, frequency, etc.) on enamel morphology in greater detail.

## Conclusion

Within the limitations of this in vitro study, the following conclusions were drawn:

1. The combined use of Er, Cr: YSGG laser and nano-hydroxyapatite (nano-HA) demonstrated superior success in the remineralization and acid resistance of initial enamel lesions compared to the individual applications of CPP-ACP + F or nano-HA.
2. Performing the laser application prior to nano-HA (G4) provided higher sustainability against acid challenges by ensuring better integration of mineral particles into the enamel surface.
3. While laser-assisted protocols dramatically increased enamel microhardness, they also resulted in an increase in surface roughness.

## Supplementary Information

Supplementary Material 1. Additional File 1 File name: Supplementary Material 1.xlsx Title of data: Raw data of microhardness and surface roughness measurements. Description of data: This file contains all individual measurements for each specimen in all experimental groups at each stage of the study.

## Data Availability

The datasets supporting the conclusions of this article are included within the article and its additional file (Supplementary Material 1).

## Abbreviations

ANOVA: Analysis of Variance
CPP-ACP: Casein Phosphopeptide-Amorphous Calcium Phosphate
CPP-ACP + F: Casein Phosphopeptide-Amorphous Calcium Phosphate with Fluoride
EDS: Energy Dispersive X-ray Spectroscopy
Er,Cr: YSGG: Erbium, Chromium: Yttrium-Scandium-Gallium-Garnet
HA: Hydroxyapatite
HSD: Honestly Significant Difference
Nano-HA: Nano-hydroxyapatite
Ra: Average Surface Roughness
SEM: Scanning Electron Microscopy
SHMP: Sodium Hexametaphosphate
SMH: Surface Microhardness
SPSS: Statistical Package for the Social Sciences
XRD: X-ray Diffraction
